# Modeling, Design, and Laboratory Testing of a Passive Friction Seismic Metamaterial Base Isolator (PFSMBI)

**DOI:** 10.3390/ma18020363

**Published:** 2025-01-15

**Authors:** Shayan Khosravi, Mohsen Amjadian

**Affiliations:** Department of Civil Engineering, University of Texas Rio Grande Valley, Edinburg, TX 78539, USA; shayan.khosravi01@utrgv.edu

**Keywords:** mechanical metamaterials, frequency bandgap, seismic isolation, passive control, friction, energy dissipation, Coulomb friction model, LuGre friction model, stick–slip motion

## Abstract

This paper focuses on the theoretical and analytical modeling of a novel seismic isolator termed the Passive Friction Mechanical Metamaterial Seismic Isolator (PFSMBI) system, which is designed for seismic hazard mitigation in multi-story buildings. The PFSMBI system consists of a lattice structure composed of a series of identical small cells interconnected by layers made of viscoelastic materials. The main function of the lattice is to shift the fundamental natural frequency of the building away from the dominant frequency of earthquake excitations by creating low-frequency bandgaps (FBGs) below 20 Hz. In this configuration, each unit cell contains an inner resonator that slides over a friction surface while it is tuned to vibrate at the fundamental natural frequency of the building. This resonance enhances the energy dissipation capacity of the PFSMBI system. After deriving the governing equations for four selected lattice configurations (i.e., Cases 1–4), a parametric study is performed to optimize the PFSMBI system for a wide range of harmonic ground motion frequencies. In this study, we examine how key parameters, such as the mass ratios of the cells and resonators, tuning frequency ratios, the number of cells, and the coefficient of friction, affect the system’s performance. The PFSMBI system is then incorporated into the dynamic model of a six-story base-isolated building to evaluate its effectiveness in reducing the floor acceleration and inter-story drift under actual earthquake ground motion records. This dynamic model is used to investigate the effect of stick–slip motion (SSM) on the energy dissipation performance of a PFSMBI system by employing the LuGre friction model. The numerical results show that the optimized PFSMBI system, through its lattice structure and frictional resonators, effectively reduces floor acceleration and inter-story drift by leveraging FBGs and frictional energy dissipation, particularly when SSM effects are properly accounted for. Finally, a small-scale prototype of the PFSMBI system with two cells is developed to verify the effect of SSM. This experimental validation highlights that neglecting SSM can lead to an overestimation of the energy dissipation performance of PFSMBI systems.

## 1. Introduction

Seismic base isolation (SBI) is an effective passive structural control method for reducing seismic demands on both load-bearing structural members and non-structural elements of multi-story buildings [1]. For this reason, it plays a key role in modern performance-based seismic design for such structures. This control method involves placing a layer of bearings made from flexible materials between the building’s base floor and the ground [2], where the low lateral stiffness of these bearings helps decouple the building from the ground during an earthquake, effectively shifting the building’s natural frequency beyond the predominant frequency of the earthquake. This frequency shift prevents the building from resonating with the earthquake [3,4]. Studies have shown that SBI can simultaneously reduce both floor acceleration and inter-story drift in buildings [3,5], resulting in maintaining the structural integrity of the building while also ensuring the safety and functionality of non-structural elements. Among various types of base isolators, curved surface sliders, commonly referred to as friction pendulum systems (FPSs), are particularly noteworthy due to their ability to combine friction damping with a restoring force mechanism, effectively enhancing the seismic performance of buildings [6,7].

However, implementing an SBI system creates a trade-off between the decrease in the acceleration and inter-story drift of the upper floors and the increase in the displacement of the base floor. More specifically, the amplified deformation of the SBI system can increase the risk of collisions of the building with surrounding moat walls or adjacent buildings, as investigated by Polycarpou and Komodromos (2010) [8]. To address this issue, the energy dissipation capacity of the SBI system can be improved in two ways. One method involves incorporating energy-dissipative materials into the bearings, such as high-damping rubber [9] and lead cores [10]. Another method is the installation of supplemental passive or semi-active dampers, such as viscous dampers [11] and friction dampers [3], in parallel with the bearings. Although effective, these methods can be costly due to the large number of components required and the associated maintenance demands.

More recently, mechanical metamaterials have emerged as a promising alternative for enhancing the frequency-shifting and energy dissipation capabilities of dynamical systems. These materials possess unique dynamic properties, such as negative stiffness [12,13,14,15], negative mass [16], and a frequency bandgap (FBG) [17,18], which make them quite effective for vibration control in civil structures [19,20]. Among these properties, the FBG [21] is particularly advantageous for SBI, as it can significantly reduce or entirely block the transmission of seismic waves within a specific frequency range [22,23]. Most research in this area has focused on the development of periodic foundations (PFs) [24,25], which leverage FBGs in mechanical metamaterials through the integration of local resonators [26,27,28,29,30]. These PFs are installed beneath buildings and are designed to be rigid enough to support the weight of the structure vertically while isolating it from ground motion horizontally. Up to now, a wide range of PFs have been proposed and studied using both numerical and experimental methods, including PFs with a concrete matrix containing rubber-coated cells around a steel core [24,25,27,29], PFs with alternating layers of rubber (or polyurethane) and concrete [28,30], and PFs with horizontally arranged rotational resonators [26].

Despite the numerous benefits that PFs and mechanical metamaterials in general offer for SBI, they suffer from some design issues, particularly in providing sufficient viscous damping for both the resonators and the overall dynamical system. In this case, friction is a viable alternative that can be integrated into the design of mechanical metamaterials. This energy dissipation mechanism offers a more cost-effective and low-maintenance solution compared to viscous damping that has traditionally been used in these materials. The key advantage of a friction-based energy dissipation mechanism is its solid-based configuration, which simplifies its implementation by avoiding common issues in a fluid-based mechanism, such as leaks and sealing problems [31]. Furthermore, a friction-based mechanism has a relatively higher energy dissipation capacity under given cyclic loads such as those imposed by earthquakes [3,32]. For this reason, in recent years, there has been growing interest in incorporating friction into both the analysis and design of mechanical metamaterials for vibration control [33,34,35,36]. These studies, however, provide limited insight into the effects of stick–slip motion (SSM) on the behavior of frictional metamaterials. SSM is a nonlinear phenomenon in which the two sliding surfaces alternately stick to each other and then slip in a repetitive cycle which causes an unwanted high-frequency jerky motion [3,37]. More specifically, during the sticking phase, the frictional metamaterial stops dissipating energy as the two sliding surfaces are locked. Therefore, by neglecting SSM, we may overestimate the performance and effectiveness of frictional metamaterials in vibration isolation, highlighting a gap in the current literature that needs to be addressed.

In this paper, a new seismic metamaterial is presented for the base isolation of multi-story buildings in which the damping of the local resonators within the cells is provided by friction. This SBI system, which is termed the Passive Friction Seismic Metamaterial Base Isolator (PFSMBI), consists of a series of interconnected cells, which are attached to each other through inner layers of viscoelastic materials. These layers, which are modeled by linear springs and viscous dampers, can be designed to shift the fundamental natural frequency of the building away from the dominant frequency of earthquake excitations. The proposed PFSMBI has a fully solid-based energy dissipation mechanism and offers a simple configuration that can be easily designed and tuned for a wide range of input excitation frequencies.

The rest of the paper is organized as follows: Section 2 describes the dynamic model of the PFSMBI system, which will be used to characterize its nonlinear hysteretic behavior using the Coulomb friction model (CFM). Section 3 presents a parametric study examining the performance of the PFSMBI system under harmonic excitations by varying the key parameters identified in Section 2. In Section 4, the LuGre friction model (LFM) [38] will be introduced to the dynamic model to account for the effects of SSM on local resonators and evaluate the efficiency of the PFSMBI system in reducing floor accelerations and inter-story drifts of a multi-story building under real earthquake records. Section 5 employs a finite element simulation to investigate the formation of FBGs for two distinct unit cell configurations, and Section 6 presents an experimental study on a small-scale prototype of the seismic metamaterial with two cells to demonstrate that SSM can downgrade the energy dissipation capability.

## 2. Dynamic Model

### 2.1. Configuration of the PFSMBI System

**Figure 1:**
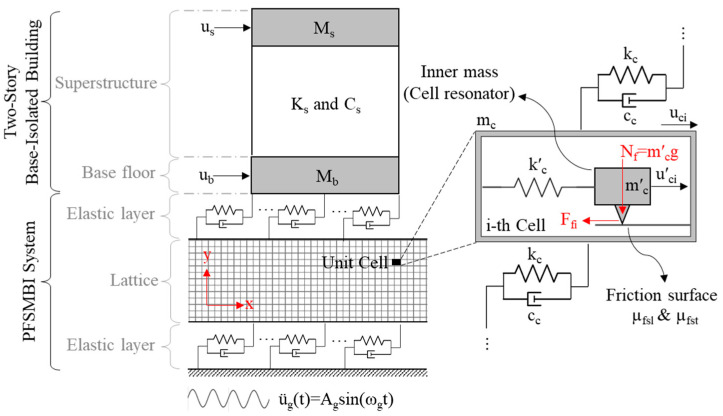
Dynamic model of a two-story base-isolated building equipped with the PFSMBI system under a harmonic ground motion.

Figure 1 illustrates the analytical model of the PFSMBI system installed on a two-story base-isolated building subjected to a horizontal ground motion along the *x*-axis, characterized by the ground acceleration ü_g_(t). In this model, M_s_, C_s_, and K_s_ denote the mass, damping, and stiffness of the superstructure, respectively, while M_b_ denotes the mass of the base floor.

As can be seen, the PFSMBI system is composed of a lattice with a periodic structure that consists of identically configured cells. These cells are interconnected through a series of viscoelastic coupling elements characterized by the stiffness and damping coefficients k_c_ and c_c_, respectively. These elements represent the elastic and damping properties of the materials used to (1) connect the unit cells to each other and (2) attach the lattice structure to both the base floor and the base floor. The primary reason for using the same material is to simplify and improve the practicality of the design. This approach has been widely adopted in the design and fabrication of metamaterials developed for vibration isolation (see references [39,40]). In such applications, the unit cells are rigid and typically constructed from steel, with rubber serving as the connecting material between them. Here, rubber is commonly chosen due to its flexibility, durability, and ease of implementation.

As shown in Figure 1, each unit cell, with a mass m_c_, contains an inner mass m′_c_ (i.e., the cell resonator) that can slide over a friction surface with the sliding and sticking friction coefficients µ_fsl_ and µ_fst_, respectively. The normal force acting on this surface is constant, assuming it to be equal to the weight of the cell resonator, which can be expressed as N_f_ = m′_c_ g. The lattice can deform along the *x*-axis while remaining rigid along the *y*-axis to support the building’s weight. As shown in the figure, the lattice is connected to the building through an elastic layer that transfers the force F_iso_ to the base floor to control the motion of the building along the *x*-axis. A similar elastic layer is also used to connect the lattice to the ground. Finally, it is assumed that the dimensions of the lattice are smaller than the input seismic wavelength, ensuring that all cells respond simultaneously to the ground motion.

In the design of the proposed PFSMBI system, we aim to achieve three key objectives: (I) reduce the absolute acceleration of the superstructure, (II) minimize the inter-story drift of the floors, and (III) enhance the energy dissipation capacity to limit the increased displacement at the base floor. The PFSMBI system has a versatile configuration, with components that can be designed for multi-objective seismic applications. Firstly, the combined stiffness of the two elastic layers and the springs between interconnected cells (i.e., the lattice flexibility in general) can be optimized to shift the building’s natural frequency away from the dominant frequency of earthquake excitations. This frequency shift is essential for reducing the risk of resonance, particularly at higher frequencies, thereby mitigating potential damage to the building. As a result, both the absolute acceleration and inter-story drift of the floors can be reduced simultaneously [3,5,41,42], addressing two key objectives (Objectives I and II) in the design of the PFSMBI system. Secondly, the cell resonators within the lattice can be tuned to resonate at the building’s natural frequency. This tuning enhances the energy dissipation capacity of the PFSMBI system, aligning with Objective III of the design. In this configuration, the cell resonators act similarly to tuned mass dampers, replicating their energy dissipation mechanism by effectively absorbing and dissipating the vibrational energy of the lattice [43,44,45].

A key aspect of the PFSMBI system design is the role of friction in controlling the motion of cell resonators. To achieve optimal performance, the friction within these cells must be precisely calibrated. If friction is too low, the resonators may behave erratically, reducing their energy dissipation capabilities. Conversely, excessive friction can result in SSM. More specifically, during the sticking phase, the resonators lock onto the friction surface, halting their relative motion and thus suspending the energy dissipation process. In addition, when the friction surface remains locked, it introduces stiffness into the system, which can lead to high-frequency responses that undermine the PFSMBI system’s overall energy dissipation efficiency. Consequently, carefully adjusting the friction is essential for maintaining smooth, controlled resonator motion.

### 2.2. Equation of Motion

**Figure 2:**
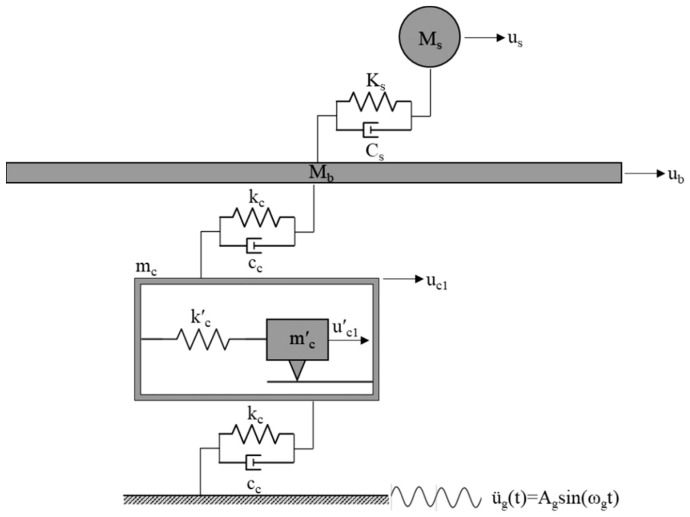
Dynamic model of the two-story base-isolated building equipped with a PFSMBI system with a single cell (N_c_ = 1).

In this section, we derive the equation of motion for the dynamic model of a two-story base-isolated building equipped with the PFSMBI system in its most basic configuration, which includes only a single cell (i.e., Case 1 with N_c_ = 1). Figure 2 shows this dynamic model. In subsequent sections, additional configurations involving multiple cells (i.e., Case 2–4) will be studied to provide a comprehensive analysis of the dynamic behavior of the PFSMBI system.

The equations of motion for the masses of the superstructure and base floor can be written as follows:

(1a) $$M_{s}{\ddot{u}}_{s}+C_{s}{\dot{u}}_{s}-{C_{s}\dot{u}}_{b}+K_{s}u_{s}-{K_{s}u}_{b}=-M_{s}{\ddot{u}}_{g}$$

(1b) $$M_{b}{\ddot{u}}_{b}+{(C}_{s}+c_{c}){\dot{u}}_{b}-C_{s}{\dot{u}}_{s}-c_{c}{\dot{u}}_{c1}{+(K}_{s}+k_{c})u_{b}-K_{s}u_{s}-k_{c}u_{c1}=-M_{b}{\ddot{u}}_{g}$$

(1c) $$m_{c}^{\prime }{\ddot{u}}_{c1}^{\prime }+k_{c}^{\prime }u_{c1}^{\prime }-k_{c}^{\prime }u_{c1}+F_{f1}=-m_{c}^{\prime }{\ddot{u}}_{g}$$

(1d) $$m_{c}{\ddot{u}}_{c1}+{\left(2c_{c}\right)\dot{u}}_{c1}-c_{c}{\dot{u}}_{b}+{\left(2k_{c}+k_{c}^{\prime }\right)u}_{c1}-k_{c}u_{b}-k_{c}^{\prime }u_{c1}^{\prime }-F_{f1}=-m_{c}{\ddot{u}}_{g}$$

where $F_{f1}$ is the friction force in Cell 1. Equations (1a)–(1d) can be written in the following matrix form:

(2a) $$M\ddot{U}(t)+C\dot{U}(t)+KU(t)+\Lambda_{f}F_{f}(t)=-M\iota_{g}{\ddot{u}}_{g}(t)$$

(2b) $$M=\left[\begin{array}{cccc} M_{s} & 0 & 0 & 0\\ 0 & M_{b} & 0 & 0\\ 0 & 0 & m_{c}^{\prime } & 0\\ 0 & 0 & 0 & m_{c} \end{array}\right],C=\left[\begin{array}{cccc} C_{s} & -C_{s} & 0 & 0\\ -C_{s} & C_{s}+c_{c} & 0 & -c_{c}\\ 0 & 0 & 0 & 0\\ 0 & -c_{c} & 0 & 2c_{c} \end{array}\right],\;and\;K=\left[\begin{array}{cccc} K_{s} & -K_{s} & 0 & 0\\ -K_{s} & K_{s}+k_{c} & 0 & -k_{c}\\ 0 & 0 & k_{c}^{\prime } & -k_{c}^{\prime }\\ 0 & -k_{c} & -k_{c}^{\prime } & 2k_{c}+k_{c}^{\prime } \end{array}\right]$$

where $U(t)={\left\{u_{s},u_{b},u_{c1}^{\prime },u_{c1}\right\}}^{T}$ is the displacement vector, $\dot{U}(t)$ and $\ddot{U}(t)$ are the relative velocity and acceleration vectors, $\iota_{g}={\left\{1,1,1,1\right\}}^{T}$ is the earthquake influence vector, $\Lambda_{f}={\left\{0,0,1,-1\right\}}^{T}$ is the friction force influence vector, and $F_{f}\left(t\right)$ is the friction force developed over the friction surface within the cell. The friction force can be computed using the Coulomb friction model [46] defined below.

(3) $$F_{fi}={\mu_{fsl}N}_{fi}sgn\left({\dot{u}}_{fi}\right)$$

where ${\dot{u}}_{fi}$ is the relative velocity between the cell resonator and the cell wall, and sgn(.) denotes the sign function. In this study, friction has been selected as the energy dissipation mechanism over traditional viscous damping due to the unique characteristics of metamaterials, particularly their compact dimensions. Implementing viscous damping in such systems poses challenges especially in a small space inside the cells, as it often requires specialized materials or fluids that may not align well with the compact and modular design of metamaterials. In contrast, friction offers a practical and straightforward solution by leveraging solid topology and easily achievable features such as joint connections. This friction model does not account for SSM. In Section 5, a more complex friction model will be explored, addressing the nonlinear behavior of SSM and its effects on the motion of the cell resonators as well as the amount of energy that they can dissipate to control the deformation of lattice [38,47,48,49].

### 2.3. Model Parameters

To evaluate the performance of the proposed PFSMBI system, the following key parameters, which control the deformation of the lattice and its energy dissipation capability, are defined. In addition, these parameters control the absolute acceleration and inter-story drift of the superstructure floor, which are critical in assessing the risk of damage to load-bearing structural members and non-structural elements.

The natural frequency and critical damping ratio of the superstructure without the PFSMBI system are defined as follows, respectively:

(4a) $$\omega_{s}=\sqrt{\frac{K_{s}}{M_{s}}}$$

(4b) $$\xi_{s}=\frac{C_{s}}{2M_{s}\omega_{s}}$$

Similarly, the natural frequency and critical damping ratio for the cell (or lattice) are defined as follows, respectively:

(5a) $$\omega_{c}=\sqrt{\frac{k_{c}}{m_{c}+m_{c}^{\prime }}}$$

(5b) $$\xi_{c}=\frac{c_{c}}{2\left(m_{c}+m_{c}^{\prime }\right)\omega_{c}}$$

It is assumed that all the cells are identical, with the same mass and a linear spring-damper system connecting them. The natural frequency of the cell resonator is also defined as follows:

(6) $$\omega_{c}^{\prime }=\sqrt{\frac{k_{c}^{\prime }}{m_{c}^{\prime }}}$$

Accordingly, for the parametric study in Section 4, the following dimensionless parameters are defined. First, the mass ratio of the lattice is given by the following:

(7) $$\mu_{c}=N_{c}\left(1+\mu_{c}^{\prime }\right)\frac{m_{c}}{M_{b}+M_{s}}$$

in which $\mu_{c}^{\prime }$ is the ratio of the cell resonator’s mass to the cell’s mass, defined as follows:

(8) $$\mu_{c}^{\prime }=\frac{m_{c}^{\prime }}{m_{c}}$$

Second, the frequency ratios of the lattice and the cell resonator to the frequency of the non-isolated building (i.e., superstructure) are defined as follows:

(9a) $$\beta_{c}=\frac{\omega_{c}}{\omega_{s}}$$

(9b) $$\beta_{c}^{\prime }=\frac{\omega_{c}^{\prime }}{\omega_{s}}$$

Finally, the ratio of the input frequency to the frequency of the non-isolated building is expressed as follows:

(10) $$\beta_{g}=\frac{\omega_{g}}{\omega_{s}}$$

### 2.4. Lattice with Four Cell Configurations

**Figure 3:**
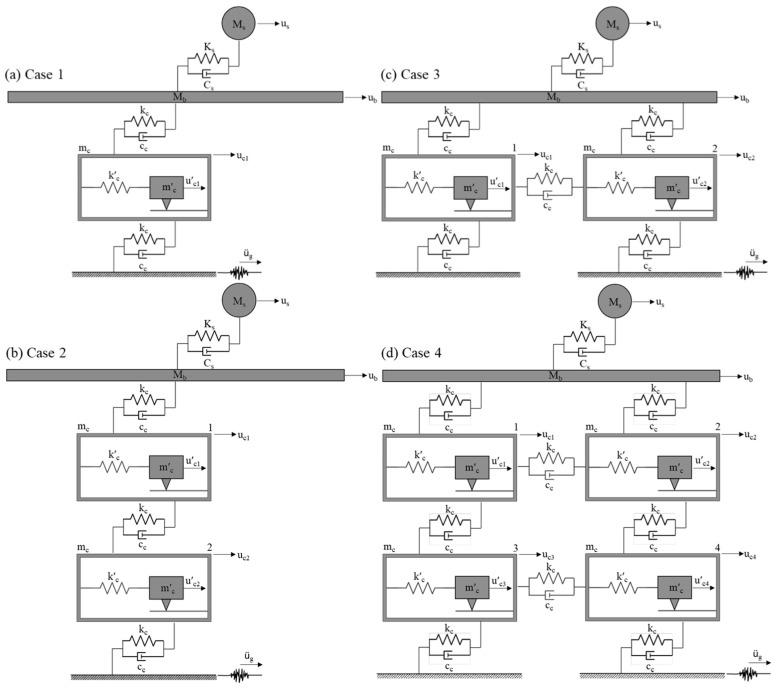
Lattice with four cell configurations: (**a**) Case 1, (**b**) Case 2, (**c**) Case 3, and (**d**) Case 4.

To accurately capture the nonlinear effects of friction within the cells on the response of the base-isolated building, a sufficiently small time-step size is used for the numerical integration (i.e., Δt ≤ 0.001 s). The effectiveness of the PFSMBI system is assessed for four different cell configurations, and each will be optimized for its respective set of parameters, introduced in the previous section. These cell configurations, shown in Figure 3, are defined as follows:

(i) Case 1 with N_c_ = 1: In this case, a single cell is positioned between the base floor of the building and the ground. This case represents the most basic form of the proposed PFMSBI system, which includes only a single cell with a resonator.
(ii) Case 2 with N_c_ = 2: This case includes two vertically aligned cells connecting the base floor to the ground. This case shows the influence of the vertical extension of cells in which the cells are acting in parallel. The cells have a layered configuration, which is like the soil layers beneath a building.
(iii) Case 3 with N_c_ = 2: In this case, two horizontally aligned cells are used to connect the base floor to the ground. This case shows the influence of the horizontal extension of cells in which the cells are acting in series.
(iv) Case 4 with N_c_ = 4: This case consists of four cells arranged in a 2 × 2 configuration, which is a combination of Case 2 and Case 3. This case is a combination of Cases 2 and 3 showing the influence of the extension of cells in both the vertical and horizontal directions.

It should be noted that the total mass of the cells remains the same in these cases.

## 3. Response to Harmonic Excitations

### 3.1. Two-Story Base-Isolated Building

To evaluate the effectiveness of the proposed PFSMBI system, it is implemented on a two-story base-isolated building model under a harmonic ground motion with a wide range of excitation frequency (see Figure 1). The two-story base-isolated building model adopted for this numerical study was originally developed by Ramallo et al. (2002) to investigate smart base isolation systems [41]. This model is a simplified dynamic representation of the six-story base-isolated building studied in Section 5 for seismic analysis. This simplification is justified as the superstructure effectively behaves as a rigid body due to the flexibility of the isolation system [2]. Table 1 lists the parameters of this model and their values.

The harmonic ground motion is characterized by the acceleration ü_g_(t) = A_g_sin(ω_g_t), where A_g_ and ω_g_ are the amplitude and frequency of the ground acceleration, respectively. It is assumed that A_g_ = 1.0 g. For friction simulation, the sliding friction coefficient is set to be $\mu_{fsl}$ = 0.25. The equation of motion of the building model with the PFSMBI system is implemented in MATLAB (R2023a) and is numerically solved by using the 4-th order Runge–Kutta method (i.e., ode45) in Simulink (R2023a) [50]. To accurately capture the nonlinear effects of friction within the cells on the response of the base-isolated building, a sufficiently small time-step size is used for the numerical integration (i.e., Δt ≤ 0.001 s). The performance of the two-story base-isolated building in these configurations will be compared to the baseline case without the PFSMBI system (N_c_ = 0). It is important to note that the total mass of the cells, in all four cases, is assumed to be equal.

### 3.2. Natural Frequencies and Modes

The natural frequency of the two-story base-isolated building is determined by solving the following equation:

(11) $$\left|K-M\omega^{2}\right|=0$$

where ω_n_ is the natural frequency of n-th mode, and the matrices **K** and **M** for Case 1 are defined by Equation (2b). The corresponding matrices for Cases 2, 3, and 4 are provided in Appendix A for brevity.

**Figure 4:**
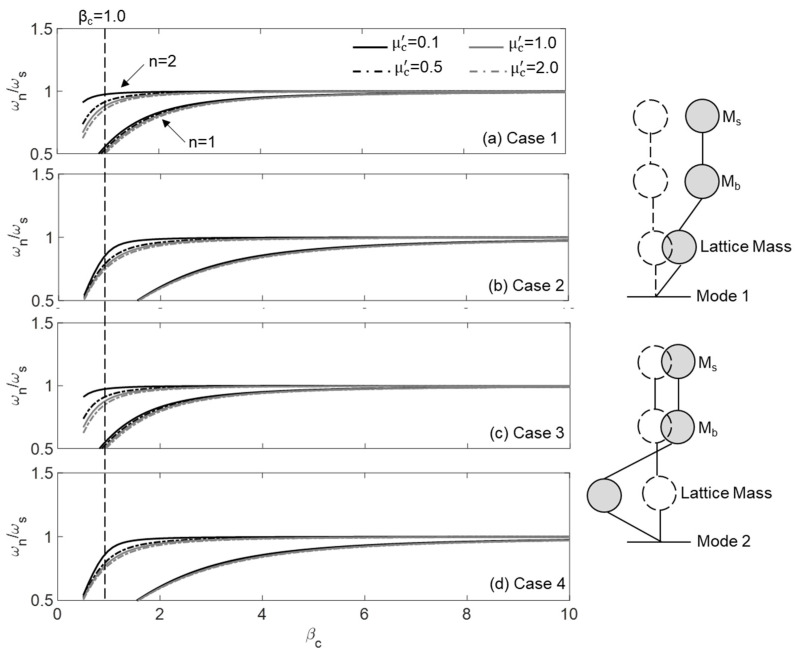
Natural frequencies of Mode 1 and Mode 2 for the base-isolated building equipped with the PFSMBI system in (**a**) Case 1, (**b**) Case 2, (**c**) Case 3, and (**d**) Case 4.

Fundamentally, only the first two modes of a base-isolated building model are significant. The first mode (*n* = 1) represents the rigid body motion of the building, which occurs due to the flexibility of the lattice, while the second mode (*n* = 2) captures the deformation within the lattice, as illustrated in Figure 4. Figure 4a–d shows the variation in the natural frequencies of these two modes for Cases 1–4, with respect to β_c_ for $\mu_{c}^{\prime }=$ 0.1, 0.5, 1.0, and 2.0. It is also assumed that $\mu_{c}=$ 1.0 and $\beta_{c}^{\prime }=1.0$. It should be noted that β_c_ governs the flexibility of the lattice and, consequently, the performance of the PFSMBI system. By adjusting this parameter, the building’s natural frequencies can be shifted to lower ranges, thereby mitigating the risk of resonance at higher frequencies. As shown in Figure 4, for small values of β_c_ (e.g., β_c_ ≤ 1.0) in all cases, the natural frequencies of Modes 1 and 2 are well separated, indicating the effectiveness of the PFSMBI system. A similar effect is observed when $\mu_{c}^{\prime }$ is increased, though the influence is not substantial. However, as β_c_ increases, the ratio ω_n_/ω_s_ for both modes tends to approach 1.0, which indicates a reduction in the effectiveness of the PFSMBI system, regardless of the value of $\mu_{c}^{\prime }$. Another notable observation from the figure is that the values of the natural frequencies for Modes 1 and 2 are similar between Cases 1 and 3, as well as between Cases 2 and 4. However, the lattice in the latter two cases is more flexible (having a lower ω_n_/ω_s_ ratio), indicating their superiority over Cases 1 and 3. A more detailed comparative study will be conducted in Section 5.

### 3.3. Parametric Study

A parametric study is conducted to evaluate the effectiveness of the PSFMSI system in Cases 1–4 under the variation in model parameters defined in Section 2. This effectiveness is measured by defining and calculating the following performance indices:

1. The peak displacement of the base floor normalized to the peak displacement of the superstructure without the PFSMBI system (N_c_ = 0).
  (12a) $$J_{1}=\frac{{max\left(\left|u_{b}\left(t\right)\right|\right)}_{N\geq 1}}{{max\left(\left|u_{s}\left(t\right)\right|\right)}_{N=0}},$$
2. The peak inter-story drift of the superstructure normalized to the peak displacement of the superstructure without the PFSMBI system.
  (12b) $$J_{2}=\frac{{max\left(\left|u_{s}\left(t\right)-u_{b}\left(t\right)\right|\right)}_{N\geq 1}}{{max\left(\left|u_{s}\left(t\right)\right|\right)}_{N=0}}$$
3. The peak absolute acceleration of the superstructure normalized to the same response of the building without the PFSMBI system.
  (12c) $$J_{3}=\frac{{max\left(\left|{\ddot{u}}_{s}\left(t\right)+{\ddot{u}}_{g}\left(t\right)\right|\right)}_{N\geq 1}}{{max\left(\left|{\ddot{u}}_{s}\left(t\right)+{\ddot{u}}_{g}\left(t\right)\right|\right)}_{N=0}}$$

In this parametric study, smaller values of these indices generally indicate better performance of the PFSMBI system.

#### 3.3.1. Influence of Tuning Frequency Ratios: $\beta_{c}$ and $\beta_{c}^{\prime }$

The tuning frequency ratios $\beta_{c}$ and $\beta_{c}^{\prime }$ control the flexibility and sensitivity of the lattice and the cell resonators to the ground motion, respectively. To assess the influence of these parameters on the effectiveness of the PFSMBI system, they are varied from 0 to 5 for the four cell configurations shown in Figure 3 (i.e., Case 1–4). For this analysis, we assume $\mu_{c}=\mu_{c}^{\prime }=1.0$ and $\beta_{g}=1.0$. Here, $\beta_{g}=1.0$ implies the critical case in which the superstructure is in resonance with the ground motion, and $\mu_{c}=1.0$ implies that the lattice has a mass equivalent to that of the building, making it a practical design consideration, as it is sufficiently heavy to support the building under the ground motion.

**Figure 5:**
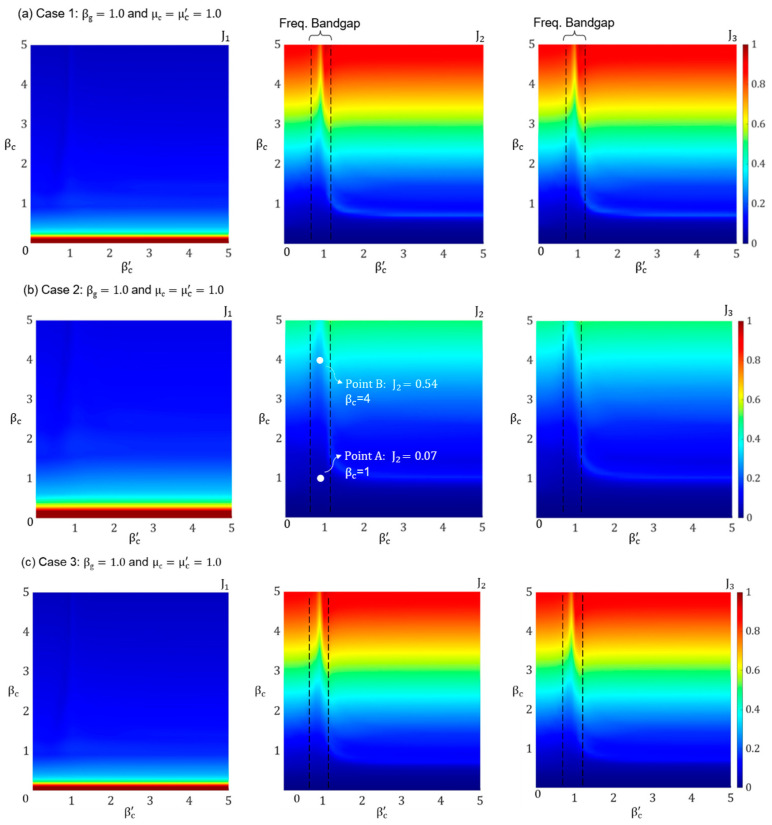

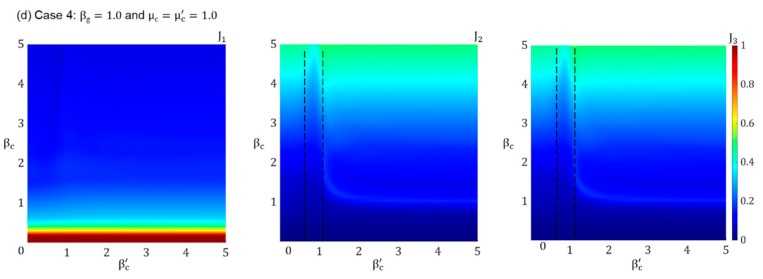
Performance indices J_1_, J_2,_ and J_3_ plotted versus $\beta_{c}$ and $\beta_{c}^{\prime }$ for (**a**) Case 1, (**b**) Case 2, (**c**) Case 3, and (**d**) Case 4.

Figure 5a–d shows the colormap plots of J_1_, J_2_, and J_3_ with respect to $\beta_{c}$ and $\beta_{c}^{\prime }$ for Cases 1–4, respectively. The vertical color bars in these plots range from 0 to 1. From these plots, it is observed that both J_2_ (i.e., the superstructure inter-story drift) and J_3_ (i.e., the superstructure absolute acceleration) show similar trends as $\beta_{c}$ and $\beta_{c}^{\prime }$ are varied. On the other hand, J_1_ (i.e., the lattice deformation) follows an opposite trend, increasing when J_2_ and J_3_ decrease. In addition, J_2_ and J_3_ remain largely unaffected by changes in $\beta_{c}^{\prime }$ except for a narrow region around $\beta_{c}^{\prime }=1$, where a resonance FBG is established. This occurs when the cell resonators are tuned to the ground motion frequency. This phenomenon is particularly advantageous when $\beta_{c}$ is large, as the resonance of the cell resonators effectively mitigates the seismic response of the superstructure.

In all four cases, as $\beta_{c}$ increases (i.e., the lattice becomes stiffer), both J_2_ and J_3_ increase, which means the overall performance of the PFSMBI system decreases. However, this increase in stiffness also decreases J_1_, meaning there is less deformation in the lattice. This trade-off suggests that while a stiffer lattice limits the deformation within the PFSMBI system, it reduces its ability to control inter-story drift and absolute acceleration in the superstructure. The best performance of the PFSMBI system for Cases 1 and 3 occurs when ${0.25<\beta }_{c}<2.5$. However, for Cases 2 and 4, the optimal performance is achieved within the broader range ${0.5<\beta }_{c}<4.0$. This indicates that the PFSMBI system in Cases 2 and 4 outperforms the other configurations, providing better action in controlling the seismic response of the superstructure.

#### 3.3.2. Influence of Mass Ratios: $\mu_{c}$, $\mu_{c}^{\prime }$

In the previous section, it was shown that the PFSMBI system with a cell configuration similar to that in Case 2 exhibits the best performance. Therefore, this configuration is selected to further investigate the influence of the mass ratios of the cells and resonators on the PFSMBI system performance at two key points, A and B, as indicated in the plots shown in Figure 5b. At these two points, the cell resonators are in resonance with the superstructure (i.e., $\beta_{c}^{\prime }=1$), but at point A, $\beta_{c}=1$ (softer lattice), and at point B, $\beta_{c}=4$ (stiffer lattice). To assess the influence of $\mu_{c}$ and $\mu_{c}^{\prime }$ on the effectiveness of the PFSMBI system, they are varied from 0 to 2, and it is assumed that $\beta_{g}=1$.

**Figure 6:**
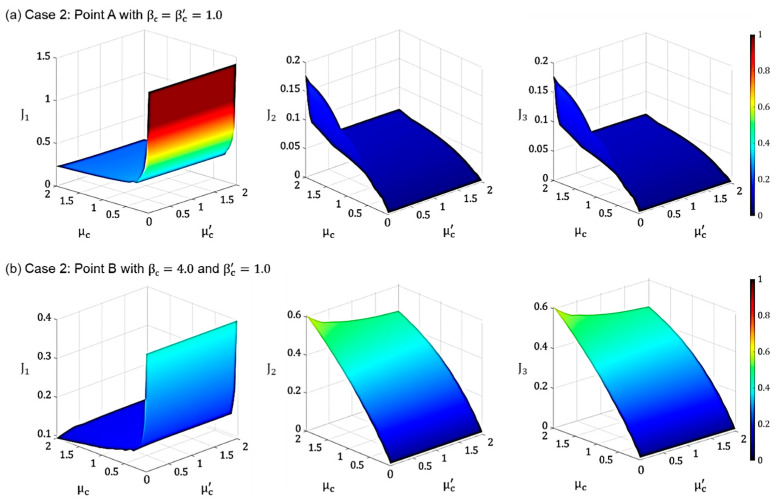
Performance indices J_1_, J_2,_ and J_3_ plotted versus $\mu_{c}$ and $\mu_{c}^{\prime }$ for Case 2 corresponding to (**a**) Point A ($\beta_{c}=1.0)$ and (**b**) Point B ($\beta_{c}=4.0)$.

Figure 6a shows the variations in the performance indices J_1_, J_2_, and J_3_ with respect to the mass ratios at Point A. It can be observed that for small values of μ_c_, the indices J_2_ and J_3_ remain relatively unchanged with respect to $\mu_{c}^{\prime }$, indicating that they are insensitive to $\mu_{c}^{\prime }$ at low values of μ_c_. However, as μ_c_ increases, improved performance (i.e., reductions in J_2_ and J_3_) is achieved when $\mu_{c}^{\prime }$ is increased. This suggests that, for a softer lattice (i.e., when β_c_ = 1), enhancing the performance requires a larger $\mu_{c}^{\prime }$. Figure 6b plots the performance indices J_1_, J_2_, and J_3_ with respect to the mass ratios at Point B, where the lattice is stiffer. Here, it is evident that the performance indices J_2_ and J_3_ are generally insensitive to $\mu_{c}^{\prime }$ regardless of the value of μ_c_. Similar to the results observed for the softer lattice in Figure 6a, it is shown that for a stiffer lattice, as in Figure 6b, decreasing μ_c_ improves the performance of the PFSMBI system. However, it is important to note that, from a practical standpoint, achieving a lattice with a μ_c_ value less than 1 may be challenging.

#### 3.3.3. Influence of Input Frequency Ratio: $\beta_{g}$

In the previous two sections, we assumed that the superstructure was in resonance with the ground motion (i.e., β_g_ = 1), which represents an extreme but uncommon loading scenario. The dominant frequency range of natural earthquakes typically spans from 0.1 Hz to 10 Hz [51]. For this reason, to provide a more comprehensive analysis of the PFSMBI system performance under a broader range of excitation frequency values, this section studies the influence of the input frequency ratio on the response of the base-isolated building. Here, the superstructure behavior is specifically analyzed at two points, A (softer lattice) and B (stiffer lattice), as discussed in Section 4.2. For this analysis, β_g_ is varied from 0 to 3, while $\mu_{c}^{\prime }$ ranges from 0 to 2, with μ_c_ set to 1.0.

**Figure 7:**
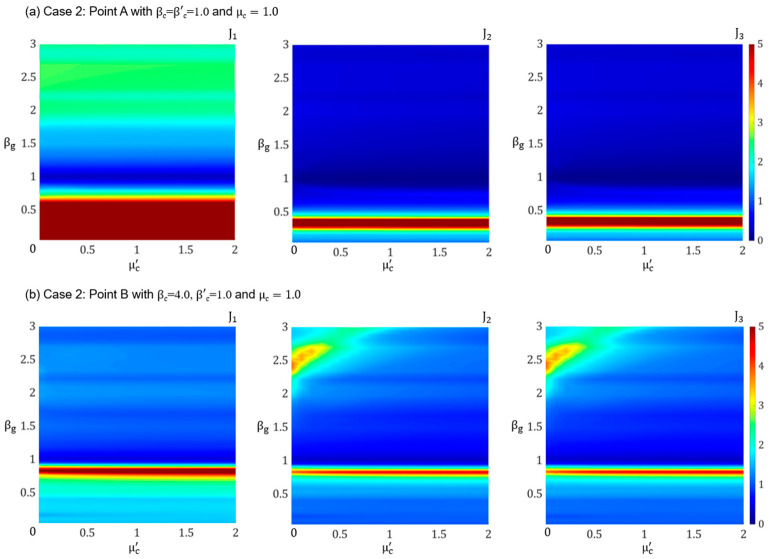
Performance indices J_1_, J_2,_ and J_3_ plotted versus $\beta_{g}$ and $\mu_{c}^{\prime }$ for Case 2 corresponding to (**a**) Point A ($\beta_{c}=1.0)$ and (**b**) Point B ($\beta_{c}=4.0)$.

From Figure 7a, it is evident that, for a softer lattice, the PFSMBI system demonstrates optimal performance for 0.90 ≤ β_g_ ≤ 1.75 when all three performance indices, J_1_, J_2_, and J_3_, remain well below 1.0. However, the system’s performance decreases significantly for β_g_ ≤ 0.75, with J_1_ increasing substantially due to the high frequency of the input excitation. The influence of $\mu_{c}^{\prime }$ (i.e., the mass damping of the cell resonators) on J_1_ is minimal; however, it can be noted that an increase in $\mu_{c}^{\prime }$ enhances the performance of the PFSMBI system, particularly when β_g_ = 1.0. For a stiffer lattice, Figure 7b indicates that the PFSMBI system achieves optimal performance when β_g_ ≥ 0.90 and $\mu_{c}^{\prime }$ > 1.0, with J_1_, J_2_, and J_3_ less than 1.0. Overall, it is observed that improved performance is correlated with an increase in $\mu_{c}^{\prime }$.

#### 3.3.4. Influence of the Number of Cells: N_c_

**Figure 8:**
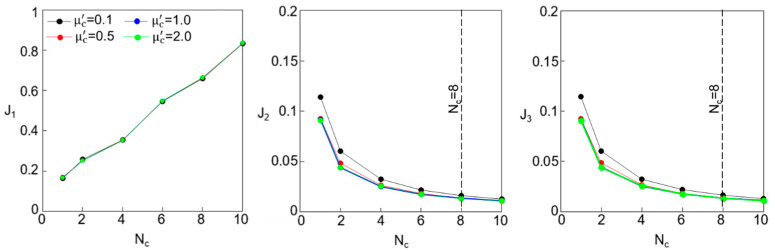
Performance indices J_1_, J_2,_ and J_3_ plotted versus N_c_ for Case 2 with $\beta_{c}=\beta_{c}^{\prime }=1.0$ and $\mu_{c}=1.0$.

The number of cells, denoted by N_c_, is a critical parameter that affects the lattice’s FBG and its energy dissipation capabilities through friction and vibration. To evaluate how N_c_ influences the effectiveness of the PFSMBI system in Case 2, the performance indices J_1_, J_2_, and J_3_ are plotted with respect to N_c_, which is varied from 1 to 10. Figure 8 shows the resulting plots for four different values of the mass ratio of the cell resonator, including $\mu_{c}^{\prime }=$ 0.1, 0.5, 1.0, and 2.0. In addition, it is assumed that $\beta_{g}=1.0$, $\beta_{c}=\beta_{c}^{\prime }=1.0$, and $\mu_{c}=1.0$. The results indicate that both J_2_ and J_3_ decrease as Nc increases; however, the rate of decrease diminishes for N_c_ values greater than 8. These reductions in the inter-story drifts (J_2_) and absolute accelerations (J_3_) of the superstructure come at the expense of an increase in J_1_, which indicates a greater deformation in the lattice as N_c_ increases. The figure also demonstrates that increasing $\mu_{c}^{\prime }$ does not affect J1 but does lead to further reductions in J_2_ and J_3_, which is due to the transfer of input kinetic energy to the resonators. It should be noted that despite this trade-off, the PFSMBI system still shows a satisfactory performance. For example, at N_c_ = 8, we obtain J_1_ = 0.65, J_2_ = 0.025, and J_3_ = 0.025, which correspond to reductions of 35%, 97.5%, and 97.5% in the base floor displacement, superstructure inter-story drift, and absolute acceleration of the two-story base-isolated building, respectively.

## 4. Response to Earthquake Excitations

### 4.1. Six-Story Base-Isolated Building Model

**Figure 9:**
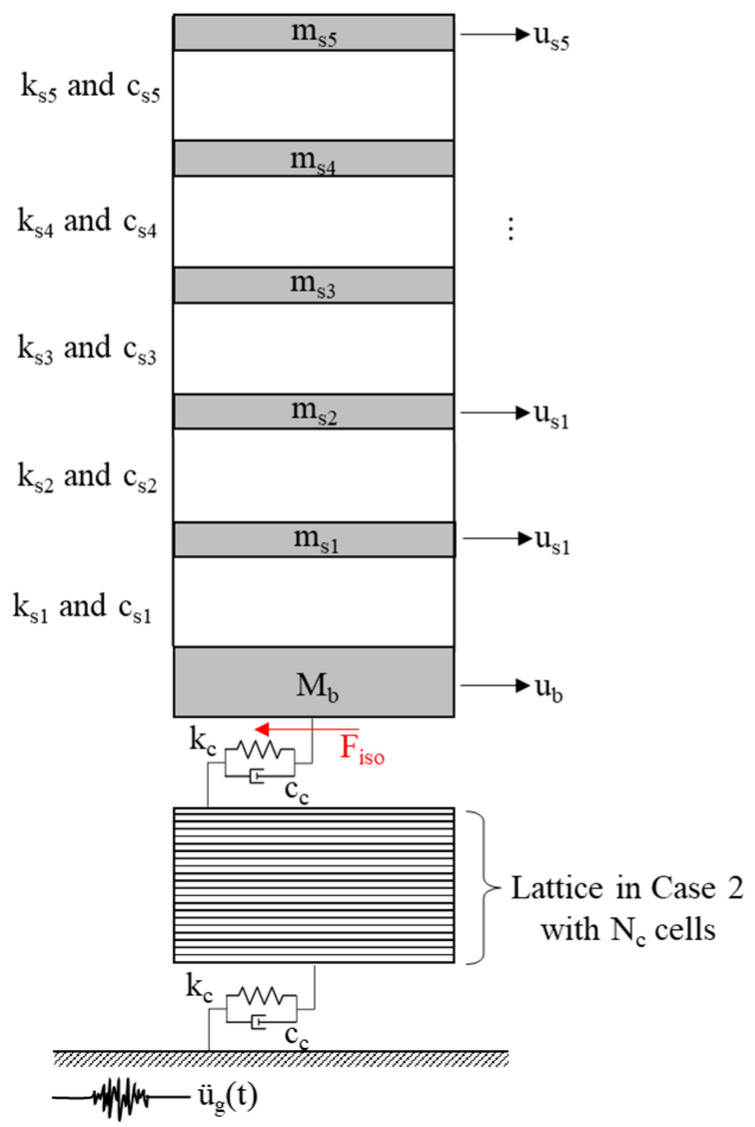
Dynamic model of a six-story base-isolated building model equipped with the PFSMBI system in Case 2.

The seismic performance of the PFSMBI system, with its lattice cells configured according to Case 2 (N_c_ = 10), is further analyzed by implementing it in the dynamic model of a multi-story base-isolated building subjected to real ground motion records, as illustrated in Figure 9. This seismic analysis enables us to assess the effectiveness of the PFSMBI system for a broader range of input frequencies and varying levels of ground acceleration intensity. The base-isolated model of a five-story building originally introduced by Kelly et al. (1987) is selected for this seismic analysis [52]. Table 2 lists the dynamic parameters of the base-isolated model of this building, including the mass, damping coefficients, and stiffness coefficients of each floor, as well as the dimensionless parameters of the PFSMBI system.

The equation of motion of the dynamic model of the multi-story base-isolated building equipped with the proposed PFSMBI system, with N_c_ = 10 (Case 2), is given by the following:

(13) $$\left[\begin{array}{cc} M_{ss} & M_{sm}\\ M_{ms} & M_{mm} \end{array}\right]\left\{\begin{array}{c} {\ddot{U}}_{s}\\ {\ddot{U}}_{m} \end{array}\right\}+\left[\begin{array}{cc} C_{ss} & C_{sm}\\ C_{ms} & C_{mm} \end{array}\right]\left\{\begin{array}{c} {\dot{U}}_{s}\\ {\dot{U}}_{m} \end{array}\right\}+\left[\begin{array}{cc} K_{ss} & K_{sm}\\ K_{ms} & K_{mm} \end{array}\right]\left\{\begin{array}{c} U_{s}\\ U_{m} \end{array}\right\}+F_{f}(t)\;=-\left[\begin{array}{cc} M_{ss} & M_{sm}\\ M_{ms} & M_{mm} \end{array}\right]\left\{\begin{array}{c} \iota_{sg}\\ \iota_{mg} \end{array}\right\}{\ddot{u}}_{g}(t)$$

where M_ss_ (6 × 6) is the mass submatrix corresponding to the base-isolated building, including both the superstructure and the base floor; M_mm_ (2 N_c_ × 2 N_c_) is the mass submatrix corresponding to the lattice; and $M_{sm}=M_{ms}^{T}$ are the mass submatrices linking the degrees of freedom of the base-isolated building to those of the cells and their resonators. In this case, these submatrices are equal to $0_{6\times 2N_{c}}$. The corresponding damping and stiffness submatrices can be defined in a similar manner using the damping and stiffness matrices presented in Appendix A. Furthermore, in Equation (13), $U_{s}$ (6 × 1) and $U_{m}$ (2 N_c_ × 1) are the displacement vectors of the floors and the lattice (i.e., both cells and their resonators), respectively; $\iota_{sg}$ and $\iota_{mg}$ are the earthquake influence vectors of the floors and the lattice; and F_f_(t) is the friction force vector.

**Figure 10:**
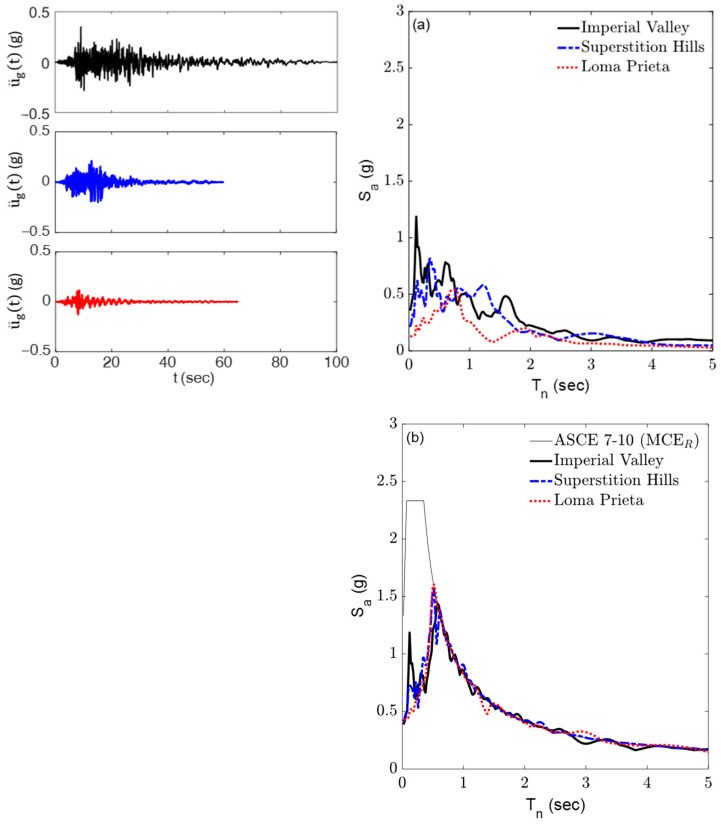
Three far-field ground acceleration records and their (**a**) unscaled and (**b**) scaled acceleration spectra for plotted ζ = 5%.

The system performance is evaluated by conducting a response history analysis (RHA) on the base-isolated building model under three selected far-field ground motion records collected in the 1979 Imperial Valley, 1987 Superstition Hills, and 1989 Loma Prieta earthquakes. These records, sourced from the Pacific Earthquake Engineering Research Center (PEER)’s strong motion database, are detailed in Table 3 [53]. To ensure consistency in the intensity levels of the ground motion records, they are scaled to match the ASCE 7-10 Maximum Considered Earthquake (MCE_R_) design spectrum, which corresponds to a return period of approximately 2475 years. This spectrum represents the seismicity of a site in California with Site Class B, a critical damping ratio of 5%, and a peak ground acceleration (PGA) of 0.876 g. The scaling process was performed for the period range T_n_ = 0.05–5 s. Figure 10 illustrates the time histories of these records, along with their scaled and unscaled acceleration spectra, plotted for a critical damping ratio of ζ = 5%.

### 4.2. LuGre Friction Model (LFM)

The friction model represented by Equation (3), commonly referred to as the CFM, does not account for SSM—a nonlinear phenomenon that naturally and inevitably arises in many dynamical systems with friction. Disregarding SSM in the analysis of the proposed PSFMSI system could lead to an overestimation of its performance under earthquake excitations [32,54]. When the resonators stick, they become locked, halting the process of energy dissipation within the PFMSFI system. This simultaneously introduces an additional stiffness into the system, which may trigger an undesirable high-frequency response in the PFSMBI system. In this section, the LFM, which effectively accounts for SSM, is used to model the friction between each cell resonator and the cell wall [38]. The friction force in the i-th cell is defined as follows:

(14a) $$F_{fi}\left(t\right)=\sigma_{0}z_{i}+\sigma_{1}{\dot{z}}_{i}$$

where $\sigma_{0}$ is the stiffness coefficient, $\sigma_{1}$ is the damping coefficient, and $z_{i}$ is the internal state variable, which is given by solving the following differential equation:

(14b) $${\dot{z}}_{i}={\dot{u}}_{fi}-\sigma_{0}\frac{\left|{\dot{u}}_{fi}\right|}{g\left({\dot{u}}_{fi}\right)}$$

In this equation, ${\dot{u}}_{fi}$ is the relative velocity, and g(.) is a function that represents the transition from the sticking phase to the sliding phase, capturing the Stribeck effect, which is defined as follows:

(14c) $$g\left({\dot{u}}_{fi}\right)=F_{fsl}+\left(F_{fst}-F_{fsl}\right)e^{{-\left(\frac{{\dot{u}}_{fi}}{v_{fs}}\right)}^{2}}$$

where $F_{fsl}=\mu_{fsl}N_{f}$ and $F_{fst}=\mu_{fst}N_{f}$, with $\mu_{fst}=1.25\mu_{fsl}$, are the sliding and sticking friction forces, respectively, and v_fs_ = 0.025 m/s is the Stribeck velocity. This small value is chosen to ensure that Stribeck effects occur at low velocities [55,56,57].

### 4.3. Energy Dissipation Performance

To assess the impact of SSM on the performance of the proposed PFSMI system, a new performance index is defined as the ratio of the area under the time-history curve of the dissipated energy due to friction to that of the input energy, as follows:

(15a) $$J_{4}=\frac{\int_{0}^{t_{g}}E_{Df}(t)dt}{\int_{0}^{t_{g}}E_{Ir}(t)dt}$$

where E_df_(t) represents the total dissipated energy due to friction within the cells and is defined as follows:

(15b) $$E_{Df}(t)=\sum_{n=1}^{N_{c}}\left[\int_{0}^{t}F_{fi}(\tau ){\dot{u}}_{fi}(\tau )d\tau \right]$$

Furthermore, in Equation (15a), E_Ir_(t) denotes the relative input energy of the base-isolated building during the earthquake, defined by the following:

(15c) $$E_{Ir}\left(t\right)=-\int_{0}^{t}{{\ddot{u}}_{g}\left(\tau \right)\iota }_{g}^{T}M\dot{U}(\tau )d\tau$$

The performance index J_4_ can accurately describe the influence of SSM on the performance of the PFSMBI system as the cell resonators stop dissipating energy during the stick phase. A higher value of J_4_ indicates a greater amount of energy dissipated by the cell resonators, which may be overestimated if SSM is not considered in modeling the friction. This can be demonstrated by calculating J_4_ using the LFM and comparing it to the corresponding values from the CFM.

Table 4 presents the values of J_4_ for β_c_ = 1.0 and 4.0, with $\mu_{c}^{\prime }$ = 0.1, 0.5, 1.0, and 2.0, assuming $\beta_{c}^{\prime }=1.0$, and $\mu_{c}=1.0$. Here, the frequency ratios β_c_ and $\beta_{c}^{\prime }$ have been normalized to the frequency of the first mode of the non-isolated building. It is observed that, for all three scaled ground motion records, the values of J_4_ calculated using the CFM are almost higher than those obtained with the LFM, indicating an overestimation of energy dissipation in the cells when SSM is disregarded by CFM. This discrepancy is more pronounced when the lattice is softer, i.e., when β_c_ = 1.0.

**Figure 11:**
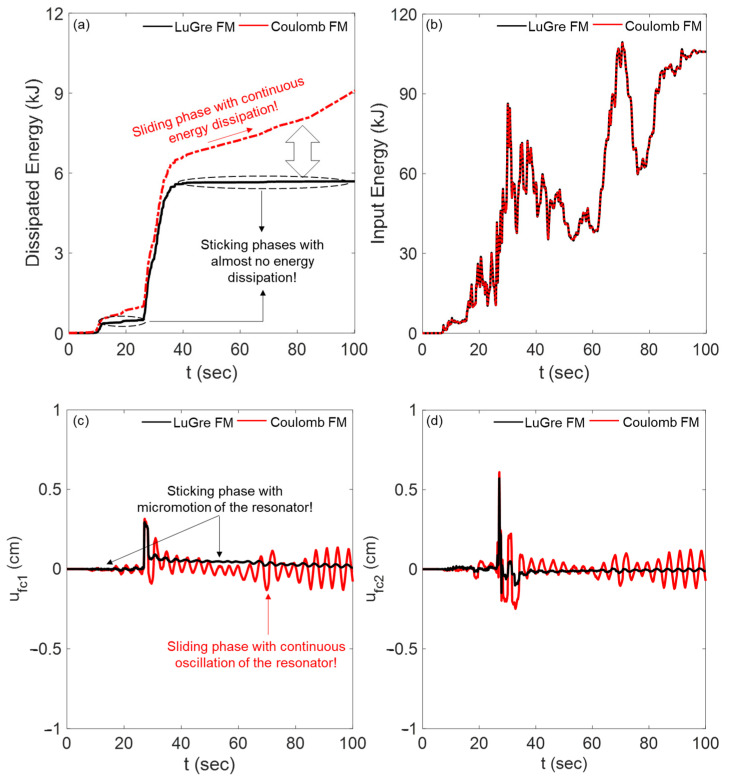
Influence of SSM on (**a**,**b**) the total dissipated energy and the total input energy within the PFSMBI system and (**c**,**d**) the relative displacement of resonators in Cells 1 and 2 for the Imperial Valley earthquake.

For further clarification, Figure 11a,b illustrates the time histories of the total energy dissipated due to friction within the cells of the PFSMBI system and the relative input energy of the base-isolated building, respectively, assuming β_c_ = 1.0. These figures compare the energy dissipation performance of the PFSMBI system for the Imperial Valley earthquake when the LFM and CFM friction models are used. A noticeable difference in energy dissipation behavior is observed between the two friction models. For the LFM, the dissipated energy evolves in a stepwise manner, remaining constant during sticking phases, when the resonators stick and stop dissipating energy. In contrast, under the CFM, the dissipated energy increases continuously during sliding phases. For instance, Figure 11a shows that the PFSMBI system undergoes two major sticking phases: one between 17 and 27 s and another between 38 and 100 s. During these intervals, the low intensity of the ground motion fails to overcome the breakaway friction force, keeping the resonators stationary and preventing energy dissipation. This behavior is not captured by the CFM, where the resonators persist in sliding phases with continuous energy dissipation. Despite these differences, Figure 11b indicates that the total input energy remains consistent for both friction models.

Furthermore, Figure 11c,d presents the time histories of the relative displacements of resonators within Cells 1 and 2, highlighting further differences between the LFM and CFM friction models in the PFSMBI system. Under the LFM, the resonators exhibit sticking behavior, with their relative displacements remaining constant except for minor micromotions. On the other hand, under the CFM, the resonators experience continuous oscillations around the origin, reflecting their persistent sliding phases.

**Figure 12:**
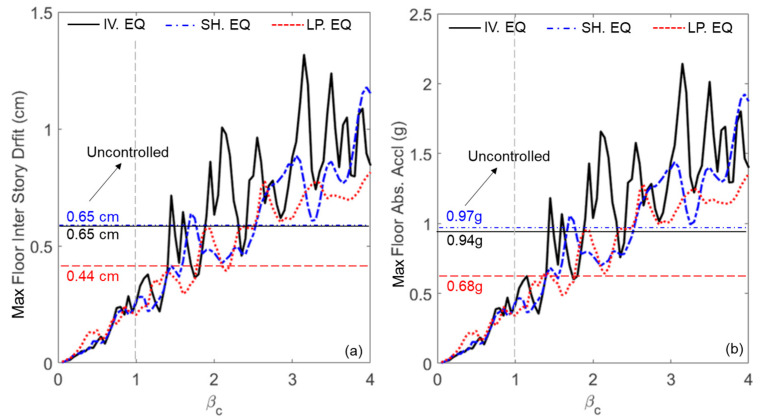
Influence of the frequency ratio of the lattice on the maximum (**a**) floor inter-story drift and (**b**) floor absolute acceleration for the three ground motion records.

Having proved that the LFM can accurately simulate friction within the cells, we now employ this model to investigate the performance of the PFSMBI system in controlling the floor’s inter-story drifts and absolute accelerations under the three ground motion records. Figure 12 shows the maximum values of these two responses plotted versus β_c_. These values are compared to the average (over the floors) values computed for the uncontrolled case (i.e., without the PFSMBI system). The results indicate that, overall, as β_c_ increases, both responses also increase. Notably, resonance occurs at specific values of β_c_, resulting in peak response values. For example, during the Imperial Valley earthquake, resonance occurs at β_c_ = 2.3, resulting in increases in both responses beyond the uncontrolled case: 0.65 cm for the inter-story drift and 0.97 g for the absolute acceleration. Furthermore, the best performance of the PFSMBI system across all three ground motion records is achieved when β_c_ < 1. This is also valid under harmonic ground motion as demonstrated in Section 4.

**Figure 13:**
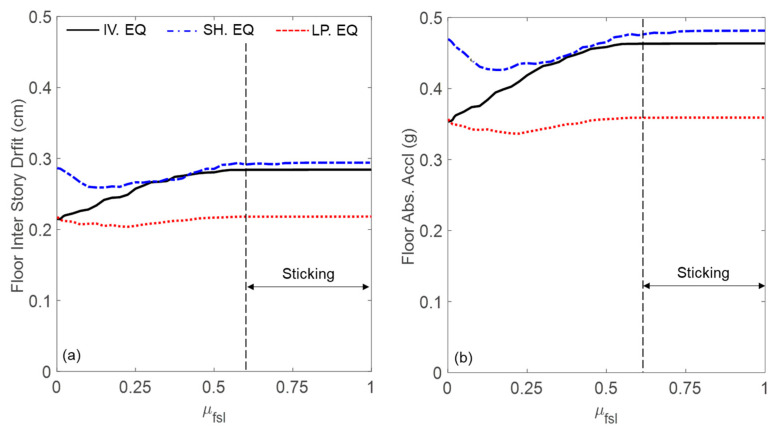
Influence of the friction coefficient on the maximum (**a**) floor inter-story drift and (**b**) floor absolute acceleration for the three ground motion records.

Similarly, Figure 13 shows the maximum floor inter-story drift and absolute acceleration plotted against the sliding friction coefficient (μ_fsl_) for the three ground motion records, assuming β_c_ = 1 (soft lattice). The results indicate that both responses increase as μ_fsl_ rises from 0 to 0.6. However, for μ_fsl_ > 0.6, these responses remain unchanged due to the increased friction force causing stick–slip behavior. In this regime, the resonators become completely stuck, effectively eliminating their oscillatory contribution to the dynamics of the system.

### 4.4. Hysteretic Behavior

**Figure 14:**
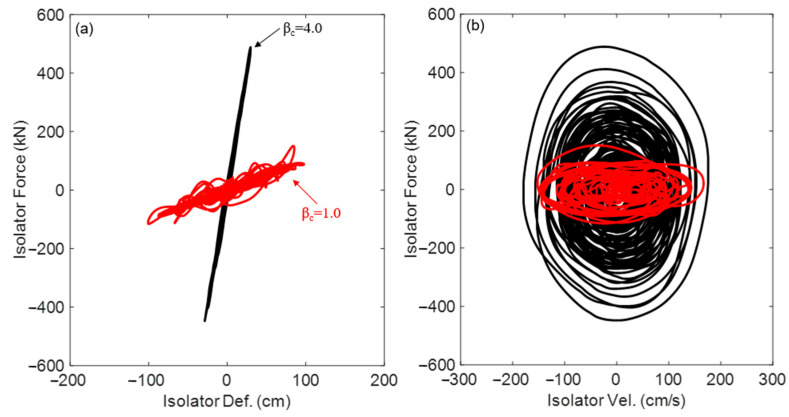
Hysteresis behavior of the proposed PFSMBI system under the Imperial Valley earthquake for β_c_ = 1.0 (red) and 4.0 (black): (**a**) force–displacement and (**b**) force–velocity curves.

The passive control force of the PFSMBI system transferred to the base floor is given by the following:

(16) $$F_{iso}\left(t\right)=k_{c1}\left[u_{b}\left(t\right)-u_{c11}\left(t\right)\right]+c_{c1}\left[{\dot{u}}_{b}\left(t\right)-{\dot{u}}_{c11}\left(t\right)\right]$$

where $u_{c11}\left(t\right)$ and ${\dot{u}}_{c11}\left(t\right)$ are, respectively, the displacement and velocity of the upper cell (i.e., cell #1) connected to the base floor. This force will be utilized to characterize the hysteretic behavior of the PFSMBI system and illustrate its hysteretic loops for a range of values of β_c_ and other key parameters described in Table 2. As an example, Figure 14a,b shows the force–displacement and force–velocity hysteretic loops of the proposed PFSMBI system, respectively, under the Imperial Valley earthquake. The hysteretic loops are compared for two cases: β_c_ = 1.0 (soft lattice) and β_c_ = 4.0 (stiff lattice). It can be observed that as β_c_ increases, indicating a stiffer lattice, the force–displacement hysteresis curve becomes more linear. In contrast, when β_c_ decreases and the lattice becomes softer, the curve exhibits greater nonlinearity. Furthermore, the force–velocity relationship of the PFSMBI system takes on an elliptical shape due to the inertial effects of the cells and their resonator masses.

## 5. Frequency Bandgap Analysis

**Figure 15:**
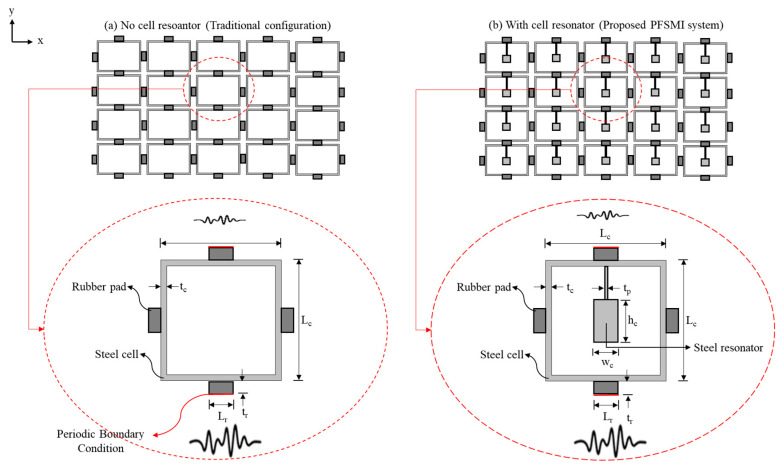
Two cell configurations: (**a**) Traditional cell configuration with no cell resonator where periodic steel cells are connected by rubber pads. (**b**) Proposed cell configuration featuring a steel resonator at the center of each unit cell (red: Periodic Boundary Condition).

In this section, the FBG properties of the proposed PFSMBI system are analyzed. The analysis is performed using the finite element method (FEM) to simulate the free vibration response of the PFSMBI system. A real-size geometry for the cells is adopted to ensure an accurate representation of the PFSMBI system performance and its bandgap characteristics. The unit cell consists of periodic square-shaped steel boxes with an outer dimension of L_c_ and thickness t_c_, along with four rubber pads on each outer side of the cell, with length L_r_ and thickness t_r_. These rubber pads are used to connect the steel boxes together and provide the restoring force between the cells. Two configurations of the unit cell are studied: (I) the traditional configuration without a cell resonator, as studied in [39] and shown in Figure 15a, and (II) the proposed configuration with a cell resonator, composed of a steel mass connected to the top of the cell wall through a thin steel sheet, as shown in Figure 15b. The thickness of the cells in the z-direction is assumed to be 25 cm. The geometrical and material properties of the cells are given in Table 5 and Table 6, respectively.

**Figure 16:**
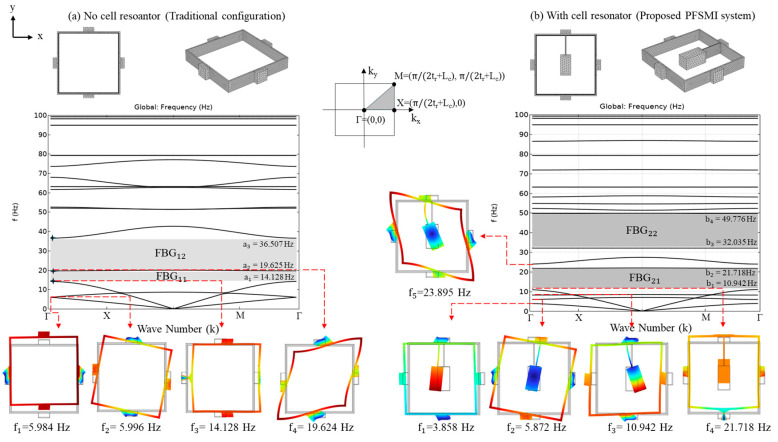
FBGs and mode shapes of the two cell configurations: (**a**) Traditional cell configuration. (**b**) Proposed cell configuration with a steel resonator.

The FE models for both cell configurations, as shown in Figure 15, are developed in COMSOL Multiphysics 6.2 [58] and meshed accordingly, as illustrated in Figure 16. A Floquet periodic boundary condition is applied to the outer surfaces of the top and bottom rubber pads along the *y*-axis, where seismic waves propagate from the lower to upper layers. This boundary condition is defined by the wave number k_x_ = k (k_y_ = k_z_ = 0). To account for the lateral continuity of motion in the cells of the PFSMBI system, as expected for the optimal cell configuration in Case 2, a continuity periodic boundary condition is imposed on the outer surfaces of the left and right rubber pads along the *x*-axis. In this simulation, friction is not taken into account, as this section merely focuses on the free vibration response of the cells.

The traditional cell configuration (type I) uses hollow steel cells with rubber pads to generate bandgaps for seismic wave attenuation. In contrast, the PFSMBI cell, with its inner steel resonator (type II), enhances local resonance effects, leading to a broader bandgap and more effective attenuation of low-frequency seismic waves (0–20 Hz). This improved design offers better protection of the superstructure against earthquakes compared to the traditional hollow cell. Figure 16a,b shows the FBGs of these two types of cell configurations, respectively. The traditional hollow unit cell shows a bandgap between 14.128 Hz and 19.625 Hz (FBG_11_), with mode shapes at critical frequencies showing displacement patterns within the hollow structure. In contrast, the PFSMBI unit cell exhibits a broader first bandgap at lower frequencies due to its steel resonator, with the first bandgap (FBG_21_) between 10.942 Hz and 21.718 Hz. This FBG can be even lowered by increasing the size of the cells. The mode shapes for the PFSMBI unit cell show more complex vibration patterns due to the steel resonator, resulting in enhanced local resonance effects that improve attenuation in the low-frequency range.

## 6. Experimental Study

**Figure 17:**
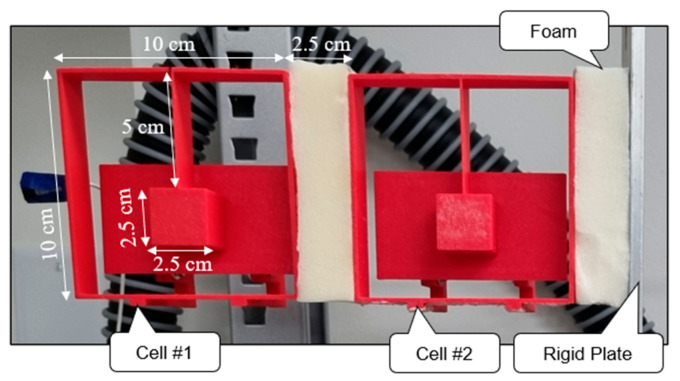
Close view of a prototype of the proposed PFSMBI system with two cells.

A small-scale prototype was fabricated using 3D printing for laboratory testing to examine the influence of SSM on the energy dissipation capability of the proposed isolation system and its effectiveness in controlling acceleration response, particularly during the sticking phase. The configuration of this small-scale prototype is based on the design outlined in Section 5, with adjustments to the size of the inner resonator masses and variations in the materials used for fabrication. The prototype, shown in Figure 17, consists of two cells (Cell 1 and Cell 2), each having the dimensions 100 × 100 × 25 mm. These cells are connected by a flexible foam layer with dimensions of 25 × 100 × 25 mm, and each one contains a resonator with the dimensions 25 × 25 × 30 mm with a mass of 23.25 g, which is attached to the top wall of the cell via a thin column with the dimensions of 1 × 50 × 25 mm, as illustrated in Figure 17. The cells and resonators are fabricated using Basic Polylactic Acid (PLA) through a 3D printing process.

**Figure 18:**
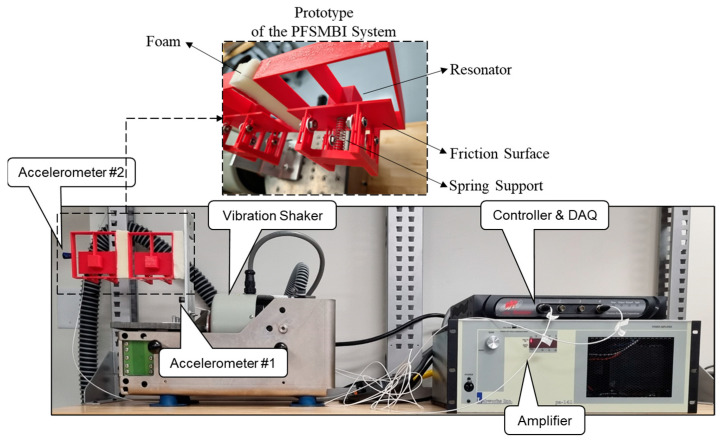
Experimental setup for testing of a prototype of the proposed PFSMBI system with two cells under a sine sweep motion.

**Figure 19:**
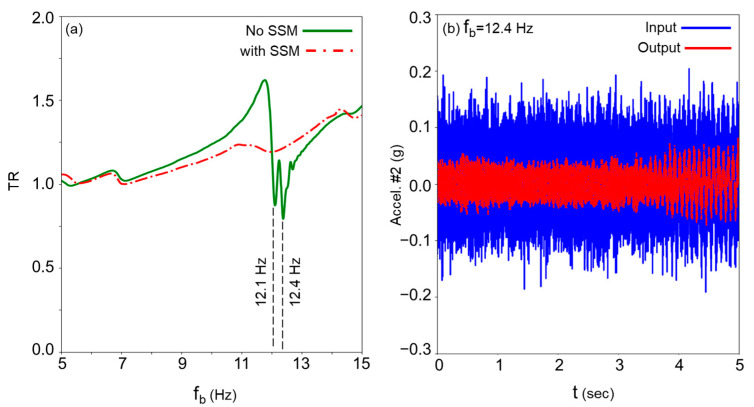
Experimental study results considering effects of SSM: (**a**) transmissibility ratio and (**b**) input vs. output acceleration for f_b_ = 12.4 Hz.

Figure 18 illustrates the experimental setup where the fabricated prototype is mounted on an Electrodynamic Shaker (Vibration Research: VR5600, 110 F-lb pk) through the rigid plate. The system is subjected to a horizontal sweeping harmonic motion within a frequency range of 5–15 Hz and a maximum acceleration of 0.15 g. The experimental setup also includes an amplifier, a controller (Vibration Research: VR9500, 4 Channels, 24-bit Resolution) for controlling the shaker and recording acceleration data in both frequency and time domains, two accelerometers (PCB, 100 mV/g sensitivity, ±50 g pk), and a computer for processing the output signals. The accelerometers employed in the experiment are designated as Accelerometer 1 and Accelerometer 2. The first accelerometer measures the acceleration at the base, serving as the input response, while the second one captures the vibration of the outer wall of Cell 1, which is referred to as the output response. As also shown in Figure 18, a spring support mechanism is incorporated to model friction within the cells. A flat thin plate is used to serve as the friction surface, which is secured to the cell using adjustable screws. This allows for varying the normal force to modify the frictional interaction as needed. In this study, two friction scenarios are analyzed: Case 1, with SSM, and Case 2, without SSM. In Case 1, representing an extreme scenario, the normal force (or friction force) is sufficiently increased to ensure that the resonators remain stuck to the friction plate throughout the test. Figure 19a shows the transmissibility ratio (TR), defined as the ratio of the output response to the input response, plotted against the input frequency range of 5–15 Hz. This figure compares the results for the two cases: with and without SSM. The results indicate that when the resonators remain in their sticking phases (with SSM), the isolation system is unable to dissipate energy effectively. In this scenario, the TR remains greater than 1, signifying an amplification of the input response rather than its reduction. Conversely, when the resonators are allowed to slide freely, the TR decreases, particularly around the frequencies where the resonators in Cells 1 and 2 resonate with the input frequency, specifically at f_b_ = 12.1 Hz and f_b_ = 12.4 Hz, respectively. This demonstrates the enhanced energy dissipation capability of the isolation system when SSM is minimized. Figure 19b compares the output acceleration versus the input acceleration for f_b_ = 12.4 Hz.

## 7. Conclusions

This paper studies a novel seismic isolator, the Passive Friction Mechanical Metamaterial Seismic Isolator (PFSMBI) system. This study is supported by comprehensive theoretical and analytical modeling to mitigate seismic hazards in multi-story buildings. Inspired by the principles of tuned mass dampers and mechanical metamaterials, the PFSMBI system employs friction as a primary mechanism for energy dissipation for the inner resonators within the cells. A detailed parametric study revealed that the performance of the PFSMBI system can be optimized by tuning critical parameters, including the mass ratios, the frequency ratios, and the number of cells. Friction proved to be a reliable source of energy dissipation, provided there is continuous sliding motion on the frictional surface. To accurately capture the stick–slip motion (SSM), the LuGre friction model (LFM) was adopted, offering a more realistic representation of frictional behavior compared to simpler models like the Coulomb friction model (CFM). Finite element (FE) analysis further demonstrated the capabilities of the PFSMBI system through a specialized topology designed to achieve low-frequency bandgaps. The analysis also highlighted the critical role of the inner resonator mass in widening these bandgaps within the low-frequency range (0–20 Hz), which is vital for attenuating seismic excitations. Furthermore, a simple experimental study was performed to demonstrate the limitations of the system due to stick–slip motion (SSM). These findings underscore the importance of optimally controlling friction to mitigate SSM and ensure effective energy dissipation. In conclusion, the PFSMBI system provides a robust and adaptable solution for enhancing multi-story buildings’ resilience against seismic activity. By combining the principles of mechanical metamaterials and friction-based damping, this innovative approach represents an innovation in seismic isolation technology. This study emphasizes the potential of metamaterial-based seismic isolators to transform traditional methods of earthquake resilience in civil engineering.

## Figures and Tables

**Table 1:** Parameters of the two-story base-isolated building model [41].

Floor Number	M_b_ (kg)	ω_c_ (rad/s)	ξ_c_ (%)
Base	6800.0	It depends on β_c_, defined by Equation (9b).	5.0
Floor Number	m_s_ (kg)	ω_s_ (rad/s)	ξ_s_ (%)
1st	29,485.0	20.1	2.0

**Table 2:** Parameters of the six-story base-isolated building model [52].

Floor Number	M_b_ (kg)	$\mu_{c}$	$\mu_{c}^{\prime }$	$\beta_{c}$	$\beta_{c}^{\prime }$	$\mu_{fsl}$
Base	6800	1.0	Var.	Var.	1.0	0.25
Floor Number	m_s_ (kg)	c_s_ (kN.s/m)	k_s_ (kN/m)			
1st	5897.0	67.0	33,732.0			
2nd	5897.0	58.0	29,093.0			
3rd	5897.0	57.0	28,621.0			
4th	5897.0	50.0	24,954.0			
5th	5897.0	38.0	19,059.0			

**Table 3:** Ground motion acceleration records used for the response history analysis.

SN ^a^	Name	Year	Magnitude (M)	Station	Component	PGA (g)	
						Unscaled	Scaled
0169	Imperial Valley (IV. EQ)	1979	6.5	Delta	DLT352	0.350	0.384
0728	Superstition Hills (SH. EQ)	1987	6.5	Westmorland Fire Station	WSM180	0.211	0.408
0757	Loma Prieta (LP. EQ)	1989	6.9	Dumbarton Bridge West End	DUMB267	0.127	0.427

^a^ RSN: Record sequence number in the PEER’s next generation attenuation (NGA) ground motion database.

**Table 4:** Values of the performance index J_4_ calculated using the LFM and CFM.

	β_c_ = 1.0 (Soft Lattice)							
Friction Model	with SSM (LuGre)				w/o SSM (Coulomb)			
$\mu_{c}^{\prime }$	0.1	0.5	1	2	0.1	0.5	1	2
Imperial Valley	0.0293	0.0657	0.0756	0.0827	0.0342	0.0785	0.1014	0.1128
Superstition Hills	0.0321	0.0731	0.0863	0.0943	0.0335	0.0834	0.1073	0.1144
Loma Prieta	0.0157	0.0342	0.0408	0.0444	0.0170	0.0433	0.0555	0.0575
	β_c_ = 4.0 (Stiff Lattice)							
Friction Model	with SSM (LuGre)				w/o SSM (Coulomb)			
$\mu_{c}^{\prime }$	0.1	0.5	1	2	0.1	0.5	1	2
Imperial Valley	0.1005	0.2886	0.3691	0.4269	0.0992	0.2890	0.3740	0.4361
Superstition Hills	0.0572	0.1944	0.2688	0.3285	0.0563	0.1928	0.2679	0.3301
Loma Prieta	0.0768	0.2425	0.3215	0.3792	0.0731	0.2386	0.3221	0.3867

**Table 5:** Geometry parameters of a unit cell.

L_c_ (cm)	L_r_ (cm)	W_c_ (cm)	h_c_ (cm)	t_c_ (cm)	t_r_ (cm)	t_p_ (cm)
150	25	12.5	25	3	10	1.25

**Table 6:** Material parameters of unit cell.

Material	Density (kg/m^3^)	Young Modulus (Pa)	Poisson’s Ratio
Steel Rubber	7850	2.01 × 10^11^	0.30
	1400	1.08 × 10^5^	0.45

## Data Availability

The data presented in this study are available on request from the corresponding author. The data sets are not publicly available due to privacy and our ongoing research.

## Appendix A

In this section, we present the mass, stiffness, and damping matrices for the dynamic models in Cases 2, 3, and 4 as shown in Figure 3.

**Case 2:** For Case 2 (N_c_ = 2), the matrices **M** and **K** are given by Equations (A1) and (A2), respectively:

(A1) $$M=\left[\begin{array}{cccccc} M_{s} & 0 & 0 & 0 & 0 & 0\\ 0 & M_{b} & 0 & 0 & 0 & 0\\ 0 & 0 & m_{c}^{\prime } & 0 & 0 & 0\\ 0 & 0 & 0 & m_{c} & 0 & 0\\ 0 & 0 & 0 & 0 & m_{c}^{\prime } & 0\\ 0 & 0 & 0 & 0 & 0 & m_{c} \end{array}\right]$$

(A2) $$K=\left[\begin{array}{cccccc} K_{s} & -K_{s} & 0 & 0 & 0 & 0\\ -K_{s} & K_{s}+k_{c} & 0 & -k_{c} & 0 & 0\\ 0 & 0 & k_{c}^{\prime } & -k_{c}^{\prime } & 0 & 0\\ 0 & -k_{c} & -k_{c}^{\prime } & 2k_{c}+k_{c}^{\prime } & 0 & -k_{c}\\ 0 & 0 & 0 & 0 & k_{c}^{\prime } & -k_{c}^{\prime }\\ 0 & 0 & 0 & -k_{c} & -k_{c}^{\prime } & 2k_{c}+k_{c}^{\prime } \end{array}\right]$$

The friction force influence vectors of Cells 1 and 2 are given as $\Lambda_{f1}=\{0,0,+1,-1,00\}$ and $\Lambda_{f2}=\left\{0,0,0,0+1,-1\right\}$, respectively.

**Case 3:** For Case 3 (N_c_ = 2), the matrices **M** and **K** are given by Equations (A3) and (A4), respectively:

(A3) $$M=\left[\begin{array}{cccccc} M_{s} & 0 & 0 & 0 & 0 & 0\\ 0 & M_{b} & 0 & 0 & 0 & 0\\ 0 & 0 & m_{c}^{\prime } & 0 & 0 & 0\\ 0 & 0 & 0 & m_{c} & 0 & 0\\ 0 & 0 & 0 & 0 & m_{c}^{\prime } & 0\\ 0 & 0 & 0 & 0 & 0 & m_{c} \end{array}\right]$$

(A4) $$K=\left[\begin{array}{cccccc} K_{s} & -K_{s} & 0 & 0 & 0 & 0\\ -K_{s} & K_{s}+{2k}_{c} & 0 & -k_{c} & 0 & -k_{c}\\ 0 & 0 & k_{c}^{\prime } & -k_{c}^{\prime } & 0 & 0\\ 0 & -k_{c} & -k_{c}^{\prime } & 3k_{c}+k_{c}^{\prime } & 0 & -k_{c}\\ 0 & 0 & 0 & 0 & k_{c}^{\prime } & -k_{c}^{\prime }\\ 0 & -k_{c} & 0 & -k_{c} & -k_{c}^{\prime } & 3k_{c}+k_{c}^{\prime } \end{array}\right]$$

The friction force influence vectors of Cells 1 and 2 are given as $\Lambda_{f1}=\{0,0,+1,-1,00\}$ and $\Lambda_{f2}=\left\{0,0,0,0+1,-1\right\}$, respectively.

**Case 4:** For Case 4 (N_c_ = 4), the matrices **M** and **K** are given by Equations (A5) and (A6), respectively:

(A5) $$M=\left[\begin{array}{cccccccccc} M_{s} & 0 & 0 & 0 & 0 & 0 & 0 & 0 & 0 & 0\\ 0 & M_{b} & 0 & 0 & 0 & 0 & 0 & 0 & 0 & 0\\ 0 & 0 & m_{c}^{\prime } & 0 & 0 & 0 & 0 & 0 & 0 & 0\\ 0 & 0 & 0 & m_{c} & 0 & 0 & 0 & 0 & 0 & 0\\ 0 & 0 & 0 & 0 & m_{c}^{\prime } & 0 & 0 & 0 & 0 & 0\\ 0 & 0 & 0 & 0 & 0 & m_{c} & 0 & 0 & 0 & 0\\ 0 & 0 & 0 & 0 & 0 & 0 & m_{c}^{\prime } & 0 & 0 & 0\\ 0 & 0 & 0 & 0 & 0 & 0 & 0 & m_{c} & 0 & 0\\ 0 & 0 & 0 & 0 & 0 & 0 & 0 & 0 & m_{c}^{\prime } & 0\\ 0 & 0 & 0 & 0 & 0 & 0 & 0 & 0 & 0 & m_{c} \end{array}\right]$$

(A6) $$K=\left[\begin{array}{cccccccccc} K_{s} & -K_{s} & 0 & 0 & 0 & 0 & 0 & 0 & 0 & 0\\ -K_{s} & K_{s}+{2k}_{c} & 0 & -k_{c} & 0 & -k_{c} & 0 & 0 & 0 & 0\\ 0 & 0 & k_{c}^{\prime } & -k_{c}^{\prime } & 0 & 0 & 0 & 0 & 0 & 0\\ 0 & -k_{c} & -k_{c}^{\prime } & 3k_{c}+k_{c}^{\prime } & 0 & -k_{c} & 0 & -k_{c} & 0 & 0\\ 0 & 0 & 0 & 0 & k_{c}^{\prime } & -k_{c}^{\prime } & 0 & 0 & 0 & 0\\ 0 & -k_{c} & 0 & -k_{c} & -k_{c}^{\prime } & 3k_{c}+k_{c}^{\prime } & 0 & 0 & 0 & -k_{c}\\ 0 & 0 & 0 & 0 & 0 & 0 & k_{c}^{\prime } & -k_{c}^{\prime } & 0 & 0\\ 0 & 0 & 0 & -k_{c} & 0 & 0 & -k_{c}^{\prime } & 3k_{c}+k_{c}^{\prime } & 0 & -k_{c}\\ 0 & 0 & 0 & 0 & 0 & 0 & 0 & 0 & k_{c}^{\prime } & -k_{c}^{\prime }\\ 0 & 0 & 0 & 0 & 0 & -k_{c} & 0 & -k_{c} & -k_{c}^{\prime } & 3k_{c}+k_{c}^{\prime } \end{array}\right]$$

The friction force influence vectors of Cells 1 and 2 are given as $\Lambda_{f1}=\{0,0,+1,-1,00\}$ and $\Lambda_{f2}=\left\{0,0,0,0+1,-1\right\}$, respectively.
